# Supplementary material for: Transmembrane Amino Acid Transporters in Shaping the Metabolic Profile of Breast Cancer Cell Lines: The Focus on Molecular Biological Subtype
Source: Curr Issues Mol Biol. 2024 Dec 25;47(1):4. doi: 10.3390/cimb47010004 (PMC11763447; doi:10.3390/cimb47010004)
Supplement: Supplementary file 1 [file cimb-47-00004-s001.zip › Dyachenko_Supplementary_1.pdf]

Luminal subtypes of Breast Cancer

Gene:	Alternative name of gene:	Chromosomal location:	Protein:
SLC1A1	EAAC1; EAAT3; hEAAC1	9p24.2	Slc 1A1

miRNA:

miR-335-5p	miR-500a-5p	miR-4713-5p	miR-2113	miR-1255a	miR-4659b-3p	miR-1237-3p
miR-96-5p	miR-629-3p	miR-6867-3p	miR-758-3p	miR-6744-5p	miR-4659a-3p	miR-6832-3p
miR-204-5p	miR-4287	miR-6868-3p	miR-6720-5p	miR-6849-3p	miR-6875-3p	miR-652-5p
miR-211-5p	miR-4469	miR-7113-3p	miR-6512-3p	miR-5586-5p	miR-4641	
miR-186-5p	miR-4685-3p	miR-5010-3p	miR-1255b-5p	miR-6835-3p	miR-1248	

Gene:	Alternative name of gene:	Chromosomal location:	Protein:
SLC1A2	GLT-1; GLT1; EAAT2; HBGT	11p13	Slc 1A2

miRNA:

miR-1324	miR-455-3p	miR-3122	miR-30b-3p	miR-6883-5p	miR-1908-5p	miR-3922-3p
miR-6776-3p	miR-501-5p	miR-450a-1-3p	miR-1273h-5p	miR-6785-5p	miR-383-3p	miR-3176
miR-497-3p	miR-500a-5p	miR-6780a-5p	miR-6788-5p	miR-4728-5p	miR-15a-3p	miR-3190-5p
miR-30e-3p	miR-548as-3p	miR-6779-5p	miR-30c-2-3p	miR-149-3p	miR-24-3p	miR-31-5p
miR-30d-3p	miR-6513-5p	miR-3689c	miR-30c-1-3p	miR-7106-5p	miR-4284	miR-200c-3p
miR-30a-3p	miR-887-5p	miR-3689b-3p	miR-7977	miR-6787-5p	miR-4743-5p	
miR-1910-5p	miR-3913-5p	miR-3689a-3p	miR-6799-5p	miR-663a	miR-564	

Gene:	Alternative name of gene:	Chromosomal location:	Protein:
SLC1A3	EAAT1; GLAST; EA6; GLAST1; GLAST-1	5p13.2	Slc 1A3

miRNA:

miR-124-3p

Gene:	Alternative name of gene:	Chromosomal location:	Protein:
SLC1A4	SATT; ASCT1; ASCT-1	2p14	Slc 1A4

miRNA:

let-7b-5p	miR-615-3p	miR-3138	miR-7114-5p	miR-140-3p	miR-4789-5p	miR-362-3p
miR-335-5p	miR-484	miR-7974	miR-3681-3p	miR-448	miR-3941	miR-329-3p
miR-124-3p	miR-4800-5p	miR-3619-3p	miR-216a-3p	miR-4643	miR-1284	miR-4801
miR-215-5p	miR-4776-5p	miR-4786-5p	miR-128-3p	miR-8485	miR-603	miR-4731-3p
miR-192-5p						

Gene:	Alternative name of gene:	Chromosomal location:	Protein:
SLC1A6	EAAT4	19p13.12	Slc 1A6

miRNA:

miR-26b-5p

Gene:	Alternative name of gene:	Chromosomal location:	Protein:
SLC1A7	EAAT5	1p32.3	Slc 1A7

miRNA:						
miR-647	miR-604	miR-6504-5p	miR-8073	miR-4793-5p	miR-6736-3p	miR-6501-3p
miR-6762-3p	miR-4660	miR-3064-5p	miR-221-5p			

Gene:	Alternative name of gene:	Chromosomal location:	Protein:
SLC6A14	ND	Xq23	Slc 6A14

miRNA:
miR-26b-5p

Gene:	Alternative name of gene:	Chromosomal location:	Protein:
SLC6A15	hv7-3; NTT73; FLJ10316; V7-3; SBAT1	12q21.31	Slc 6A15

miRNA:		
miR-196b-5p	miR-23b-3p	miR-23a-3p

Gene:	Alternative name of gene:	Chromosomal location:	Protein:
SLC7A1	CAT-1; HCAT1; REC1L	3q12.3	Slc 7A1

miRNA:						
miR-16-5p	miR-3978	miR-1251-3p	miR-7852-3p	miR-181c-5p	miR-4283	miR-654-5p
miR-122-5p	miR-4694-3p	miR-4784	miR-3668	miR-181d-5p	miR-4417	miR-657
miR-125b-5p	miR-4802-3p	miR-3194-5p	miR-191-5p	miR-181b-5p	miR-4489	miR-6756-5p
miR-652-3p	miR-551b-5p	miR-3150b-3p	miR-6079	miR-181a-5p	miR-4492	miR-6766-5p
miR-155-5p	miR-3677-5p	miR-4756-3p	miR-6828-5p	miR-1205	miR-4498	miR-6804-5p
miR-124-3p	miR-4435	miR-3919	miR-5589-3p	miR-1587	miR-4537	miR-6829-5p
miR-24-3p	miR-5693	miR-3916	miR-4786-3p	miR-185-3p	miR-4656	miR-762
miR-671-5p	miR-BART4-5p	miR-6876-5p	miR-4717-3p	miR-2467-3p	miR-4675	
miR-328-3p	miR-6739-3p	miR-4476	miR-32-5p	miR-3158-5p	miR-4731-5p	
miR-92a-3p	miR-375	miR-5680	miR-92b-3p	miR-3620-5p	miR-4741	
miR-1260b	miR-6858-5p	miR-651-3p	miR-26b-5p	miR-3665	miR-5001-5p	
miR-3688-3p	miR-4689	miR-3148	miR-26a-5p	miR-3678-3p	miR-541-3p	

Gene:	Alternative name of gene:	Chromosomal location:	Protein:
SLC7A2	CAT-2; HCAT2; SLC7A2A; SLC7A2B; CAT-2A; CAT-2B	8p22	Slc 7A2

miRNA:						
miR-9-5p	miR-124-5p	miR-4658	miR-8081	miR-5007-3p	miR-4446-5p	miR-205-5p
miR-192-5p	miR-410-3p	miR-4758-5p	miR-6853-3p	miR-499a-3p	miR-5006-3p	miR-6866-3p
miR-101-3p	miR-545-5p	miR-6790-5p	miR-1206	miR-27b-3p	miR-4755-5p	miR-188-5p
miR-24-3p	miR-574-5p	miR-7849-3p	miR-144-3p	miR-27a-3p	miR-4666b	
miR-423-5p	miR-1238-5p	miR-29b-2-5p	miR-582-5p	miR-513a-5p	miR-5011-3p	
miR-30c-2-3p	miR-4255	miR-6790-3p	miR-651-5p	miR-598-3p	miR-452-3p	
miR-615-3p	miR-3659	miR-3120-3p	miR-3117-3p	miR-3680-3p	miR-3124-3p	

<table>
<tr><th>Gene:</th><th>Alternative name of gene:</th><th colspan="2">Chromosomal location:</th><th colspan="3">Protein:</th></tr>
<tr><td>SLC7A5</td><td>LAT1; E16; D16S469E; MPE16; CD98</td><td colspan="2">16q24.2</td><td colspan="3">Slc 7A5</td></tr>
<tr><th colspan="7">miRNA:</th></tr>
<tr><td>miR-626</td><td>miR-4302</td><td>miR-6728-5p</td><td>miR-6129</td><td>miR-33b-5p</td><td>miR-4755-3p</td><td>miR-6762-3p</td></tr>
<tr><td>miR-7-5p</td><td>miR-708-5p</td><td>miR-6780a-5p</td><td>miR-4510</td><td>miR-3612</td><td>miR-4781-5p</td><td>miR-6773-5p</td></tr>
<tr><td>miR-663a</td><td>miR-3139</td><td>miR-6779-5p</td><td>miR-4419a</td><td>miR-3619-5p</td><td>miR-4796-3p</td><td>miR-6777-5p</td></tr>
<tr><td>miR-126-3p</td><td>miR-28-5p</td><td>miR-3689c</td><td>miR-5186</td><td>miR-3661</td><td>miR-4797-5p</td><td>miR-6778-3p</td></tr>
<tr><td>miR-193b-3p</td><td>miR-6854-5p</td><td>miR-3689b-3p</td><td>miR-6757-5p</td><td>miR-3664-3p</td><td>miR-484</td><td>miR-6780b-5p</td></tr>
<tr><td>miR-16-5p</td><td>miR-5197-3p</td><td>miR-3689a-3p</td><td>miR-5096</td><td>miR-3689d</td><td>miR-485-5p</td><td>miR-6781-5p</td></tr>
<tr><td>miR-296-3p</td><td>miR-1295a</td><td>miR-30b-3p</td><td>miR-29b-2-5p</td><td>miR-383-3p</td><td>miR-5000-3p</td><td>miR-6787-5p</td></tr>
<tr><td>miR-671-5p</td><td>miR-8059</td><td>miR-1273h-5p</td><td>miR-140-3p</td><td>miR-3929</td><td>miR-5189-5p</td><td>miR-6791-3p</td></tr>
<tr><td>miR-193a-3p</td><td>miR-4471</td><td>miR-6799-5p</td><td>miR-6823-5p</td><td>miR-3934-3p</td><td>miR-5195-5p</td><td>miR-6794-5p</td></tr>
<tr><td>miR-1226-5p</td><td>miR-1292-5p</td><td>miR-5187-5p</td><td>miR-513b-3p</td><td>miR-3937</td><td>miR-542-3p</td><td>miR-6813-5p</td></tr>
<tr><td>miR-194-3p</td><td>miR-3135a</td><td>miR-6883-5p</td><td>miR-195-5p</td><td>miR-4264</td><td>miR-548ag</td><td>miR-6816-5p</td></tr>
<tr><td>miR-5693</td><td>miR-5008-5p</td><td>miR-6785-5p</td><td>miR-15b-5p</td><td>miR-4267</td><td>miR-548ai</td><td>miR-6820-5p</td></tr>
<tr><td>miR-205-3p</td><td>miR-4260</td><td>miR-4728-5p</td><td>miR-15a-5p</td><td>miR-4270</td><td>miR-548ba</td><td>miR-6821-5p</td></tr>
<tr><td>miR-4531</td><td>miR-1227-5p</td><td>miR-149-3p</td><td>miR-1237-5p</td><td>miR-4271</td><td>miR-548s</td><td>miR-6825-5p</td></tr>
<tr><td>miR-3911</td><td>miR-3189-3p</td><td>miR-7106-5p</td><td>miR-1264</td><td>miR-4419b</td><td>miR-5698</td><td>miR-6828-5p</td></tr>
<tr><td>miR-7847-3p</td><td>miR-6876-5p</td><td>miR-8052</td><td>miR-1273g-3p</td><td>miR-4435</td><td>miR-5700</td><td>miR-6829-3p</td></tr>
<tr><td>miR-223-3p</td><td>miR-4476</td><td>miR-3199</td><td>miR-1285-3p</td><td>miR-4436a</td><td>miR-570-5p</td><td>miR-6829-5p</td></tr>
<tr><td>miR-4697-3p</td><td>miR-30e-5p</td><td>miR-92b-5p</td><td>miR-132-5p</td><td>miR-4441</td><td>miR-586</td><td>miR-6836-3p</td></tr>
<tr><td>miR-504-3p</td><td>miR-30d-5p</td><td>miR-4515</td><td>miR-181a-2-3p</td><td>miR-4443</td><td>miR-588</td><td>miR-6846-3p</td></tr>
<tr><td>miR-5703</td><td>miR-30c-5p</td><td>miR-6875-5p</td><td>miR-185-3p</td><td>miR-4459</td><td>miR-604</td><td>miR-6851-5p</td></tr>
<tr><td>miR-4516</td><td>miR-30b-5p</td><td>miR-3126-5p</td><td>miR-1908-5p</td><td>miR-4478</td><td>miR-6085</td><td>miR-6853-5p</td></tr>
<tr><td>miR-4434</td><td>miR-30a-5p</td><td>miR-6506-5p</td><td>miR-214-3p</td><td>miR-4481</td><td>miR-6087</td><td>miR-6860</td></tr>
<tr><td>miR-184</td><td>miR-5196-5p</td><td>miR-619-5p</td><td>miR-219b-5p</td><td>miR-4488</td><td>miR-612</td><td>miR-6870-5p</td></tr>
<tr><td>miR-574-5p</td><td>miR-4747-5p</td><td>miR-4285</td><td>miR-22-3p</td><td>miR-4530</td><td>miR-6165</td><td>miR-6877-5p</td></tr>
<tr><td>miR-4475</td><td>miR-4668-5p</td><td>miR-3192-5p</td><td>miR-24-1-5p</td><td>miR-4650-5p</td><td>miR-625-5p</td><td>miR-6882-3p</td></tr>
<tr><td>miR-551b-5p</td><td>miR-7155-3p</td><td>miR-122-5p</td><td>miR-24-2-5p</td><td>miR-4663</td><td>miR-631</td><td>miR-6884-5p</td></tr>
<tr><td>miR-548c-3p</td><td>miR-6797-5p</td><td>miR-7977</td><td>miR-296-5p</td><td>miR-4690-5p</td><td>miR-647</td><td>miR-6889-5p</td></tr>
<tr><td>miR-3611</td><td>miR-3136-3p</td><td>miR-493-3p</td><td>miR-3065-3p</td><td>miR-4697-5p</td><td>miR-6509-3p</td><td>miR-6890-3p</td></tr>
<tr><td>miR-1260b</td><td>miR-1249-5p</td><td>miR-4691-3p</td><td>miR-3154</td><td>miR-4700-3p</td><td>miR-650</td><td>miR-7111-5p</td></tr>
<tr><td>miR-1260a</td><td>miR-3148</td><td>miR-449b-3p</td><td>miR-3155a</td><td>miR-4701-5p</td><td>miR-6512-3p</td><td>miR-7160-5p</td></tr>
<tr><td>miR-765</td><td>miR-3202</td><td>miR-598-3p</td><td>miR-3155b</td><td>miR-4704-3p</td><td>miR-6515-5p</td><td>miR-761</td></tr>
<tr><td>miR-658</td><td>miR-7515</td><td>miR-186-5p</td><td>miR-3179</td><td>miR-4706</td><td>miR-665</td><td>miR-7851-3p</td></tr>
<tr><td>miR-6499-3p</td><td>miR-7160-3p</td><td>miR-3133</td><td>miR-3180-3p</td><td>miR-4711-5p</td><td>miR-6720-5p</td><td>miR-8085</td></tr>
<tr><td>miR-769-5p</td><td>miR-9500</td><td>miR-6734-5p</td><td>miR-3180</td><td>miR-4716-3p</td><td>miR-6721-5p</td><td>miR-873-5p</td></tr>
<tr><td>miR-6786-3p</td><td>miR-5584-5p</td><td>miR-1255b-5p</td><td>miR-3187-3p</td><td>miR-4722-5p</td><td>miR-6724-5p</td><td></td></tr>
<tr><td>miR-4734</td><td>miR-4779</td><td>miR-1255a</td><td>miR-3187-5p</td><td>miR-4723-5p</td><td>miR-6731-5p</td><td></td></tr>
<tr><td>miR-532-3p</td><td>miR-6891-5p</td><td>miR-6133</td><td>miR-3196</td><td>miR-4725-3p</td><td>miR-6754-5p</td><td></td></tr>
<tr><td>miR-1539</td><td>miR-3173-3p</td><td>miR-6130</td><td>miR-338-3p</td><td>miR-4745-5p</td><td>miR-6755-5p</td><td></td></tr>
<tr><td>miR-1224-3p</td><td>miR-6834-5p</td><td>miR-6127</td><td>miR-33a-5p</td><td>miR-4749-5p</td><td>miR-6760-5p</td><td></td></tr>
</table>

Gene:	Alternative name of gene:	Chromosomal location:	Protein:

SLC7A7	y+LAT-1; Y+LAT1		14q11.2		Slc 7A7	
miRNA:						
miR-196a-5p	miR-3689f	miR-3689b-5p	miR-6804-3p	miR-338-3p	miR-3065-3p	miR-5586-5p
miR-5701	miR-3689e	miR-3689a-5p	miR-4679	miR-4530		

Gene:	Alternative name of gene:		Chromosomal location:		Protein:	
SLC7A8	LPI-PC1; LAT2		14q11.2		Slc 7A8	
miRNA:						
miR-185-5p	miR-4306	miR-4428	miR-4644			

Gene:	Alternative name of gene:		Chromosomal location:		Protein:	
SLC7A11	xCT		4q28.3		Slc 7A11	
miRNA:						
miR-122-5p	miR-3913-3p	miR-489-3p	miR-574-5p	miR-6826-5p	miR-520g-3p	miR-302a-3p
miR-30a-5p	miR-1277-5p	miR-25-3p	miR-3163	miR-498	miR-512-3p	miR-93-5p
miR-148b-3p	miR-5011-5p	miR-367-3p	miR-3941	miR-4279	miR-4640-3p	miR-526b-3p
miR-340-5p	miR-1279	miR-92a-3p	miR-4789-3p	miR-4532	miR-5683	miR-519d-3p
miR-155-5p	miR-595	miR-363-3p	miR-603	miR-1247-3p	miR-520e	miR-20b-5p
miR-128-3p	miR-6867-5p	miR-92b-3p	miR-362-3p	miR-5571-5p	miR-520d-3p	miR-20a-5p
miR-215-5p	miR-4789-5p	miR-190a-3p	miR-329-3p	miR-6778-3p	miR-520c-3p	miR-17-5p
miR-181a-5p	miR-186-3p	miR-223-5p	miR-8485	miR-1281	miR-520b	miR-106b-5p
miR-192-5p	miR-548e-5p	miR-410-3p	miR-6829-3p	miR-5089-5p	miR-520a-3p	miR-106a-5p
miR-32-5p	miR-6835-3p	miR-548t-5p	miR-6791-3p	miR-5589-5p	miR-373-3p	miR-6504-3p
miR-26b-5p	miR-142-3p	miR-548az-5p	miR-1976	miR-4731-5p	miR-372-3p	miR-3653-5p
miR-27a-3p	miR-500a-3p	miR-6874-5p	miR-6747-3p	miR-6506-5p	miR-302e	
miR-19a-3p	miR-767-5p	miR-505-5p	miR-6727-3p	miR-619-5p	miR-302d-3p	
miR-19b-3p	miR-5589-3p	miR-1-5p	miR-4722-3p	miR-150-5p	miR-302c-3p	
miR-218-5p	miR-4282	miR-587	miR-5193	miR-520h	miR-302b-3p	
