# Supplementary material for: Transmembrane Amino Acid Transporters in Shaping the Metabolic Profile of Breast Cancer Cell Lines: The Focus on Molecular Biological Subtype
Source: Curr Issues Mol Biol. 2024 Dec 25;47(1):4. doi: 10.3390/cimb47010004 (PMC11763447; doi:10.3390/cimb47010004)
Supplement: Supplementary file 1 [file cimb-47-00004-s001.zip › Dyachenko_Supplementary_2.pdf]

_____HER2+ subtype of Breast Cancer____

Gene:	Alternative name of gene:	Chromosomal location:	Protein:
SLC1A5	AAAT; ASCT2	19q13.32	Slc 1A5

miRNA:						
miR-193b-3p	miR-1224-3p	miR-4507	miR-6499-3p	miR-6504-3p	miR-20b-5p	miR-4728-5p
miR-155-5p	miR-1295b-3p	miR-3940-5p	miR-367-5p	miR-3159	miR-20a-5p	miR-149-3p
miR-125a-5p	miR-15a-3p	miR-4436b-5p	miR-1267	miR-562	miR-17-5p	miR-7106-5p
miR-34a-5p	miR-4717-5p	miR-23b-5p	miR-2681-3p	miR-150-5p	miR-106b-5p	miR-3926
miR-16-5p	miR-6807-5p	miR-23a-5p	miR-3664-5p	miR-433-3p	miR-106a-5p	miR-6847-3p
miR-1226-3p	miR-7151-3p	miR-24-3p	miR-4313	miR-4454	miR-6821-5p	miR-6811-3p
miR-324-3p	miR-5095	miR-4284	miR-6742-3p	miR-7107-5p	miR-6513-5p	miR-214-5p
miR-331-3p	miR-4252	miR-6774-5p	miR-6791-5p	miR-1234-3p	miR-887-5p	miR-383-3p
miR-15b-5p	miR-4742-3p	miR-635	miR-4292	miR-186-3p	miR-3913-5p	miR-330-3p
miR-5698	miR-3130-3p	miR-4772-3p	miR-8057	miR-520h	miR-3122	miR-4690-5p
miR-1976	miR-6849-3p	miR-1304-3p	miR-640	miR-520g-3p	miR-450a-1-3p	miR-552-3p
miR-3653-5p	miR-1273g-3p	miR-6890-3p	miR-378a-5p	miR-520e	miR-6780a-5p	miR-1227-3p
miR-619-5p	miR-500b-3p	miR-891a-3p	miR-6821-3p	miR-520d-3p	miR-6779-5p	miR-3199
miR-3135b	miR-4438	miR-4430	miR-6790-3p	miR-520c-3p	miR-3689c	miR-365a-5p
miR-218-5p	miR-4639-5p	miR-3652	miR-3934-5p	miR-520b	miR-3689b-3p	miR-365b-5p
miR-6746-3p	miR-6780a-3p	miR-122-5p	miR-764	miR-520a-3p	miR-3689a-3p	miR-455-5p
miR-6856-3p	miR-6852-3p	miR-504-3p	miR-125a-3p	miR-373-3p	miR-30b-3p	miR-4715-3p
miR-4762-3p	miR-3909	miR-2276-3p	miR-143-5p	miR-372-3p	miR-1273h-5p	miR-8052
miR-3192-3p	miR-590-3p	miR-4638-5p	miR-498	miR-302e	miR-6788-5p	
miR-3156-3p	miR-137	miR-1307-3p	miR-4279	miR-302d-3p	miR-30c-2-3p	
miR-1260b	miR-589-3p	miR-6741-3p	miR-6778-3p	miR-302c-3p	miR-30c-1-3p	
miR-1260a	miR-6767-3p	miR-5693	miR-6747-3p	miR-302b-3p	miR-6514-3p	
miR-188-3p	miR-8485	miR-744-3p	miR-6727-3p	miR-302a-3p	miR-7977	
miR-5704	miR-8064	miR-4423-5p	miR-4722-3p	miR-93-5p	miR-6799-5p	
miR-29a-5p	miR-6729-3p	miR-6501-5p	miR-6814-5p	miR-526b-3p	miR-6883-5p	
miR-3920	miR-6839-3p	miR-3622b-5p	miR-5697	miR-519d-3p	miR-6785-5p	

Gene:	Alternative name of gene:	Chromosomal location:	Protein:
SLC7A5	LAT1; E16; D16S469E; MPE16; CD98	16q24.2	Slc 7A5

miRNA:						
miR-626	miR-4302	miR-6728-5p	miR-6129	miR-33b-5p	miR-4755-3p	miR-6762-3p
miR-7-5p	miR-708-5p	miR-6780a-5p	miR-4510	miR-3612	miR-4781-5p	miR-6773-5p
miR-663a	miR-3139	miR-6779-5p	miR-4419a	miR-3619-5p	miR-4796-3p	miR-6777-5p
miR-126-3p	miR-28-5p	miR-3689c	miR-5186	miR-3661	miR-4797-5p	miR-6778-3p
miR-193b-3p	miR-6854-5p	miR-3689b-3p	miR-6757-5p	miR-3664-3p	miR-484	miR-6780b-5p
miR-16-5p	miR-5197-3p	miR-3689a-3p	miR-5096	miR-3689d	miR-485-5p	miR-6781-5p
miR-296-3p	miR-1295a	miR-30b-3p	miR-29b-2-5p	miR-383-3p	miR-5000-3p	miR-6787-5p
miR-671-5p	miR-8059	miR-1273h-5p	miR-140-3p	miR-3929	miR-5189-5p	miR-6791-3p
miR-193a-3p	miR-4471	miR-6799-5p	miR-6823-5p	miR-3934-3p	miR-5195-5p	miR-6794-5p
miR-1226-5p	miR-1292-5p	miR-5187-5p	miR-513b-3p	miR-3937	miR-542-3p	miR-6813-5p
miR-194-3p	miR-3135a	miR-6883-5p	miR-195-5p	miR-4264	miR-548ag	miR-6816-5p

miR-5693	miR-5008-5p	miR-6785-5p	miR-15b-5p	miR-4267	miR-548ai	miR-6820-5p
miR-205-3p	miR-4260	miR-4728-5p	miR-15a-5p	miR-4270	miR-548ba	miR-6821-5p
miR-4531	miR-1227-5p	miR-149-3p	miR-1237-5p	miR-4271	miR-548s	miR-6825-5p
miR-3911	miR-3189-3p	miR-7106-5p	miR-1264	miR-4419b	miR-5698	miR-6828-5p
miR-7847-3p	miR-6876-5p	miR-8052	miR-1273g-3p	miR-4435	miR-5700	miR-6829-3p
miR-223-3p	miR-4476	miR-3199	miR-1285-3p	miR-4436a	miR-570-5p	miR-6829-5p
miR-4697-3p	miR-30e-5p	miR-92b-5p	miR-132-5p	miR-4441	miR-586	miR-6836-3p
miR-504-3p	miR-30d-5p	miR-4515	miR-181a-2-3p	miR-4443	miR-588	miR-6846-3p
miR-5703	miR-30c-5p	miR-6875-5p	miR-185-3p	miR-4459	miR-604	miR-6851-5p
miR-4516	miR-30b-5p	miR-3126-5p	miR-1908-5p	miR-4478	miR-6085	miR-6853-5p
miR-4434	miR-30a-5p	miR-6506-5p	miR-214-3p	miR-4481	miR-6087	miR-6860
miR-184	miR-5196-5p	miR-619-5p	miR-219b-5p	miR-4488	miR-612	miR-6870-5p
miR-574-5p	miR-4747-5p	miR-4285	miR-22-3p	miR-4530	miR-6165	miR-6877-5p
miR-4475	miR-4668-5p	miR-3192-5p	miR-24-1-5p	miR-4650-5p	miR-625-5p	miR-6882-3p
miR-551b-5p	miR-7155-3p	miR-122-5p	miR-24-2-5p	miR-4663	miR-631	miR-6884-5p
miR-548c-3p	miR-6797-5p	miR-7977	miR-296-5p	miR-4690-5p	miR-647	miR-6889-5p
miR-3611	miR-3136-3p	miR-493-3p	miR-3065-3p	miR-4697-5p	miR-6509-3p	miR-6890-3p
miR-1260b	miR-1249-5p	miR-4691-3p	miR-3154	miR-4700-3p	miR-650	miR-7111-5p
miR-1260a	miR-3148	miR-449b-3p	miR-3155a	miR-4701-5p	miR-6512-3p	miR-7160-5p
miR-765	miR-3202	miR-598-3p	miR-3155b	miR-4704-3p	miR-6515-5p	miR-761
miR-658	miR-7515	miR-186-5p	miR-3179	miR-4706	miR-665	miR-7851-3p
miR-6499-3p	miR-7160-3p	miR-3133	miR-3180-3p	miR-4711-5p	miR-6720-5p	miR-8085
miR-769-5p	miR-9500	miR-6734-5p	miR-3180	miR-4716-3p	miR-6721-5p	miR-873-5p
miR-6786-3p	miR-5584-5p	miR-1255b-5p	miR-3187-3p	miR-4722-5p	miR-6724-5p	
miR-4734	miR-4779	miR-1255a	miR-3187-5p	miR-4723-5p	miR-6731-5p	
miR-532-3p	miR-6891-5p	miR-6133	miR-3196	miR-4725-3p	miR-6754-5p	
miR-1539	miR-3173-3p	miR-6130	miR-338-3p	miR-4745-5p	miR-6755-5p	
miR-1224-3p	miR-6834-5p	miR-6127	miR-33a-5p	miR-4749-5p	miR-6760-5p	

Gene:	Alternative name of gene:	Chromosomal location:	Protein:
SLC7A7	y+LAT-1; Y+LAT1	14q11.2	Slc 7A7

miRNA:						
miR-196a-5p	miR-3689f	miR-3689b-5p	miR-6804-3p	miR-338-3p	miR-3065-3p	miR-5586-5p
miR-5701	miR-3689e	miR-3689a-5p	miR-4679	miR-4530		

Gene:	Alternative name of gene:	Chromosomal location:	Protein:
SLC7A8	LPI-PC1; LAT2	14q11.2	Slc 7A8

miRNA:			
miR-185-5p	miR-4306	miR-4428	miR-4644

Gene:	Alternative name of gene:	Chromosomal location:	Protein:
SLC7A9	BAT1	19q13.11	Slc 7A9

miRNA:	
miR-335-5p	miR-142-3p

Gene:	Alternative name of gene:	Chromosomal location:	Protein:
SLC7A10	asc-1	19q13.11	Slc 7a9
**miRNA:**			
ND			

Gene:	Alternative name of gene:	Chromosomal location:	Protein:
SLC38A2	SAT2; ATA2; KIAA1382; SNAT2	12q13.11	Slc 38A2

miRNA:

miR-30a-5p	miR-3145-3p	miR-301b-3p	miR-1296-3p	miR-148a-3p	miR-1269b	miR-3912-5p
miR-335-5p	miR-4789-5p	miR-4295	miR-4801	miR-455-3p	miR-1269a	miR-548at-5p
miR-124-3p	miR-599	miR-3666	miR-4731-3p	miR-5590-3p	miR-4515	miR-26b-5p
miR-101-3p	miR-4704-5p	miR-6857-5p	miR-212-3p	miR-142-5p	miR-1288-3p	miR-26a-5p
miR-16-5p	miR-1245b-3p	miR-6878-3p	miR-132-3p	miR-4429	miR-4740-5p	miR-181d-5p
let-7b-5p	miR-19a-3p	miR-491-5p	miR-340-5p	miR-320d	miR-4711-5p	miR-181c-5p
miR-18a-3p	miR-19b-3p	miR-4450	miR-199b-5p	miR-320c	miR-6857-3p	miR-181b-5p
miR-193b-3p	miR-130a-3p	miR-6744-3p	miR-199a-5p	miR-320b	miR-4682	miR-181a-5p
let-7e-5p	miR-301a-3p	miR-4757-5p	miR-1825	miR-320a	miR-2355-3p	
miR-3161	miR-130b-3p	miR-194-3p	miR-152-3p	miR-8081	miR-4646-3p	
miR-9-3p	miR-454-3p	miR-4699-3p	miR-148b-3p	miR-4642	miR-4474-3p	

Gene:	Alternative name of gene:	Chromosomal location:	Protein:
SLC38A5	SN2; JM24; SNAT5	Xp11.23	Slc 38A5

miRNA:

miR-16-5p	miR-1281	miR-4691-5p	miR-6878-3p	miR-6855-5p	miR-3661	miR-6890-5p
miR-155-5p	miR-6875-3p	miR-4778-3p	miR-6744-3p	miR-3170	miR-3922-3p	
miR-124-3p	miR-6881-3p	miR-877-3p	miR-4757-5p	miR-193b-5p	miR-3176	
miR-30a-5p	miR-6749-3p	miR-660-3p	miR-6829-3p	miR-492	miR-2116-3p	
let-7b-5p	miR-6792-3p	miR-5193	miR-6791-3p	miR-631	miR-4733-3p	
