# Supplementary material for: Transmembrane Amino Acid Transporters in Shaping the Metabolic Profile of Breast Cancer Cell Lines: The Focus on Molecular Biological Subtype
Source: Curr Issues Mol Biol. 2024 Dec 25;47(1):4. doi: 10.3390/cimb47010004 (PMC11763447; doi:10.3390/cimb47010004)
Supplement: Supplementary file 1 [file cimb-47-00004-s001.zip › Dyachenko_Supplementary_3.pdf]

Triple Negative Breast Cancer

Gene:	Alternative name of gene:	Chromosomal location:	Protein:
SLC1A1	EAAC1; EAAT3; hEAAC1	9p24.2	Slc 1A1

miRNA:

miR-335-5p	miR-500a-5p	miR-4713-5p	miR-2113	miR-1255a	miR-4659b-3p	miR-1237-3p
miR-96-5p	miR-629-3p	miR-6867-3p	miR-758-3p	miR-6744-5p	miR-4659a-3p	miR-6832-3p
miR-204-5p	miR-4287	miR-6868-3p	miR-6720-5p	miR-6849-3p	miR-6875-3p	miR-652-5p
miR-211-5p	miR-4469	miR-7113-3p	miR-6512-3p	miR-5586-5p	miR-4641	
miR-186-5p	miR-4685-3p	miR-5010-3p	miR-1255b-5p	miR-6835-3p	miR-1248	

Gene:	Alternative name of gene:	Chromosomal location:	Protein:
SLC1A5	AAAT; ASCT2	19q13.32	Slc 1A5

miRNA:

miR-193b-3p	miR-1224-3p	miR-4507	miR-6499-3p	miR-6504-3p	miR-20b-5p	miR-4728-5p
miR-155-5p	miR-1295b-3p	miR-3940-5p	miR-367-5p	miR-3159	miR-20a-5p	miR-149-3p
miR-125a-5p	miR-15a-3p	miR-4436b-5p	miR-1267	miR-562	miR-17-5p	miR-7106-5p
miR-34a-5p	miR-4717-5p	miR-23b-5p	miR-2681-3p	miR-150-5p	miR-106b-5p	miR-3926
miR-16-5p	miR-6807-5p	miR-23a-5p	miR-3664-5p	miR-433-3p	miR-106a-5p	miR-6847-3p
miR-1226-3p	miR-7151-3p	miR-24-3p	miR-4313	miR-4454	miR-6821-5p	miR-6811-3p
miR-324-3p	miR-5095	miR-4284	miR-6742-3p	miR-7107-5p	miR-6513-5p	miR-214-5p
miR-331-3p	miR-4252	miR-6774-5p	miR-6791-5p	miR-1234-3p	miR-887-5p	miR-383-3p
miR-15b-5p	miR-4742-3p	miR-635	miR-4292	miR-186-3p	miR-3913-5p	miR-330-3p
miR-5698	miR-3130-3p	miR-4772-3p	miR-8057	miR-520h	miR-3122	miR-4690-5p
miR-1976	miR-6849-3p	miR-1304-3p	miR-640	miR-520g-3p	miR-450a-1-3p	miR-552-3p
miR-3653-5p	miR-1273g-3p	miR-6890-3p	miR-378a-5p	miR-520e	miR-6780a-5p	miR-1227-3p
miR-619-5p	miR-500b-3p	miR-891a-3p	miR-6821-3p	miR-520d-3p	miR-6779-5p	miR-3199
miR-3135b	miR-4438	miR-4430	miR-6790-3p	miR-520c-3p	miR-3689c	miR-365a-5p
miR-218-5p	miR-4639-5p	miR-3652	miR-3934-5p	miR-520b	miR-3689b-3p	miR-365b-5p
miR-6746-3p	miR-6780a-3p	miR-122-5p	miR-764	miR-520a-3p	miR-3689a-3p	miR-455-5p
miR-6856-3p	miR-6852-3p	miR-504-3p	miR-125a-3p	miR-373-3p	miR-30b-3p	miR-4715-3p
miR-4762-3p	miR-3909	miR-2276-3p	miR-143-5p	miR-372-3p	miR-1273h-5p	miR-8052
miR-3192-3p	miR-590-3p	miR-4638-5p	miR-498	miR-302e	miR-6788-5p	
miR-3156-3p	miR-137	miR-1307-3p	miR-4279	miR-302d-3p	miR-30c-2-3p	
miR-1260b	miR-589-3p	miR-6741-3p	miR-6778-3p	miR-302c-3p	miR-30c-1-3p	
miR-1260a	miR-6767-3p	miR-5693	miR-6747-3p	miR-302b-3p	miR-6514-3p	
miR-188-3p	miR-8485	miR-744-3p	miR-6727-3p	miR-302a-3p	miR-7977	
miR-5704	miR-8064	miR-4423-5p	miR-4722-3p	miR-93-5p	miR-6799-5p	
miR-29a-5p	miR-6729-3p	miR-6501-5p	miR-6814-5p	miR-526b-3p	miR-6883-5p	
miR-3920	miR-6839-3p	miR-3622b-5p	miR-5697	miR-519d-3p	miR-6785-5p	

Gene:	Alternative name of gene:	Chromosomal location:	Protein:
SLC3A1	CSNU1; D2H; RBAT; ATR1; NBAT	2p21	Slc 3A1

miRNA:

miR-382-5p	miR-548e-5p

Gene:	Alternative name of gene:	Chromosomal location:	Protein:
SLC6A4	5-HTT; SERT1	17q11.2	Slc 6a4

miRNA:

miR-335-5p	miR-1184	miR-7156-3p	miR-186-3p	miR-520e	miR-302a-3p	miR-141-5p
miR-16-5p	miR-3158-5p	miR-937-5p	miR-3934-5p	miR-520d-3p	miR-93-5p	miR-3150a-3p

miR-135a-5p	miR-4685-3p	miR-7151-3p	miR-6890-3p	miR-520c-3p	miR-526b-3p	miR-3175
miR-544a	miR-4287	miR-5095	miR-520h	miR-520b	miR-519d-3p	miR-491-5p
miR-3678-3p	miR-1237-3p	miR-5589-5p	miR-520g-3p	miR-520a-3p	miR-20b-5p	miR-6756-5p
miR-6749-3p	miR-5088-3p	miR-4731-5p	miR-6504-3p	miR-373-3p	miR-20a-5p	miR-6763-5p
miR-6792-3p	miR-5590-3p	miR-5702	miR-512-3p	miR-372-3p	miR-17-5p	miR-6766-5p
miR-4691-5p	miR-142-5p	miR-6506-5p	miR-509-5p	miR-302e	miR-106b-5p	miR-6825-5p
miR-2467-3p	miR-1264	miR-619-5p	miR-509-3-5p	miR-302d-3p	miR-106a-5p	miR-6878-5p
miR-17-3pэ	miR-6507-3p	miR-3130-3p	miR-4418	miR-302c-3p	miR-24-3p	miR-92a-2-5p
miR-1205	miR-5089-5p	miR-150-5p	miR-552-3p	miR-302b-3p	miR-1343-5p	miR-939-5p

Gene:	Alternative name of gene:		Chromosomal location:		Protein:	
SLC6A9	GLYT1; GlyT-1		1p34.1		Slc 6A9	
miRNA:						
miR-335-5p	miR-103a-3p	miR-887-5p	miR-450a-1-3p	miR-3616-3p	miR-6735-5p	miR-4436b-3p
miR-221-3p	miR-26b-5p	miR-3913-5p	miR-5196-5p	miR-7843-5p	miR-4632-5p	
miR-7-5p	miR-6513-5p	miR-3122	miR-4747-5p	miR-6879-5p	miR-4463	

Gene:	Alternative name of gene:		Chromosomal location:		Protein:	
SLC7A1	CAT-1; HCAT1; REC1L		3q12.3		Slc 7A1	
miRNA:						
miR-16-5p	miR-3978	miR-1251-3p	miR-7852-3p	miR-181c-5p	miR-4283	miR-654-5p
miR-122-5p	miR-4694-3p	miR-4784	miR-3668	miR-181d-5p	miR-4417	miR-657
miR-125b-5p	miR-4802-3p	miR-3194-5p	miR-191-5p	miR-181b-5p	miR-4489	miR-6756-5p
miR-652-3p	miR-551b-5p	miR-3150b-3p	miR-6079	miR-181a-5p	miR-4492	miR-6766-5p
miR-155-5p	miR-3677-5p	miR-4756-3p	miR-6828-5p	miR-1205	miR-4498	miR-6804-5p
miR-124-3p	miR-4435	miR-3919	miR-5589-3p	miR-1587	miR-4537	miR-6829-5p
miR-24-3p	miR-5693	miR-3916	miR-4786-3p	miR-185-3p	miR-4656	miR-762
miR-671-5p	miR-BART4-5p	miR-6876-5p	miR-4717-3p	miR-2467-3p	miR-4675	
miR-328-3p	miR-6739-3p	miR-4476	miR-32-5p	miR-3158-5p	miR-4731-5p	
miR-92a-3p	miR-375	miR-5680	miR-92b-3p	miR-3620-5p	miR-4741	
miR-1260b	miR-6858-5p	miR-651-3p	miR-26b-5p	miR-3665	miR-5001-5p	
miR-3688-3p	miR-4689	miR-3148	miR-26a-5p	miR-3678-3p	miR-541-3p	

Gene:	Alternative name of gene:		Chromosomal location:		Protein:	
SLC7A5	LAT1; E16; D16S469E; MPE16; CD98		16q24.2		Slc 7A5	
miRNA:						
miR-626	miR-4302	miR-6728-5p	miR-6129	miR-33b-5p	miR-4755-3p	miR-6762-3p
miR-7-5p	miR-708-5p	miR-6780a-5p	miR-4510	miR-3612	miR-4781-5p	miR-6773-5p
miR-663a	miR-3139	miR-6779-5p	miR-4419a	miR-3619-5p	miR-4796-3p	miR-6777-5p
miR-126-3p	miR-28-5p	miR-3689c	miR-5186	miR-3661	miR-4797-5p	miR-6778-3p
miR-193b-3p	miR-6854-5p	miR-3689b-3p	miR-6757-5p	miR-3664-3p	miR-484	miR-6780b-5p
miR-16-5p	miR-5197-3p	miR-3689a-3p	miR-5096	miR-3689d	miR-485-5p	miR-6781-5p
miR-296-3p	miR-1295a	miR-30b-3p	miR-29b-2-5p	miR-383-3p	miR-5000-3p	miR-6787-5p
miR-671-5p	miR-8059	miR-1273h-5p	miR-140-3p	miR-3929	miR-5189-5p	miR-6791-3p
miR-193a-3p	miR-4471	miR-6799-5p	miR-6823-5p	miR-3934-3p	miR-5195-5p	miR-6794-5p
miR-1226-5p	miR-1292-5p	miR-5187-5p	miR-513b-3p	miR-3937	miR-542-3p	miR-6813-5p
miR-194-3p	miR-3135a	miR-6883-5p	miR-195-5p	miR-4264	miR-548ag	miR-6816-5p
miR-5693	miR-5008-5p	miR-6785-5p	miR-15b-5p	miR-4267	miR-548ai	miR-6820-5p
miR-205-3p	miR-4260	miR-4728-5p	miR-15a-5p	miR-4270	miR-548ba	miR-6821-5p
miR-4531	miR-1227-5p	miR-149-3p	miR-1237-5p	miR-4271	miR-548s	miR-6825-5p

miR-3911	miR-3189-3p	miR-7106-5p	miR-1264	miR-4419b	miR-5698	miR-6828-5p
miR-7847-3p	miR-6876-5p	miR-8052	miR-1273g-3p	miR-4435	miR-5700	miR-6829-3p
miR-223-3p	miR-4476	miR-3199	miR-1285-3p	miR-4436a	miR-570-5p	miR-6829-5p
miR-4697-3p	miR-30e-5p	miR-92b-5p	miR-132-5p	miR-4441	miR-586	miR-6836-3p
miR-504-3p	miR-30d-5p	miR-4515	miR-181a-2-3p	miR-4443	miR-588	miR-6846-3p
miR-5703	miR-30c-5p	miR-6875-5p	miR-185-3p	miR-4459	miR-604	miR-6851-5p
miR-4516	miR-30b-5p	miR-3126-5p	miR-1908-5p	miR-4478	miR-6085	miR-6853-5p
miR-4434	miR-30a-5p	miR-6506-5p	miR-214-3p	miR-4481	miR-6087	miR-6860
miR-184	miR-5196-5p	miR-619-5p	miR-219b-5p	miR-4488	miR-612	miR-6870-5p
miR-574-5p	miR-4747-5p	miR-4285	miR-22-3p	miR-4530	miR-6165	miR-6877-5p
miR-4475	miR-4668-5p	miR-3192-5p	miR-24-1-5p	miR-4650-5p	miR-625-5p	miR-6882-3p
miR-551b-5p	miR-7155-3p	miR-122-5p	miR-24-2-5p	miR-4663	miR-631	miR-6884-5p
miR-548c-3p	miR-6797-5p	miR-7977	miR-296-5p	miR-4690-5p	miR-647	miR-6889-5p
miR-3611	miR-3136-3p	miR-493-3p	miR-3065-3p	miR-4697-5p	miR-6509-3p	miR-6890-3p
miR-1260b	miR-1249-5p	miR-4691-3p	miR-3154	miR-4700-3p	miR-650	miR-7111-5p
miR-1260a	miR-3148	miR-449b-3p	miR-3155a	miR-4701-5p	miR-6512-3p	miR-7160-5p
miR-765	miR-3202	miR-598-3p	miR-3155b	miR-4704-3p	miR-6515-5p	miR-761
miR-658	miR-7515	miR-186-5p	miR-3179	miR-4706	miR-665	miR-7851-3p
miR-6499-3p	miR-7160-3p	miR-3133	miR-3180-3p	miR-4711-5p	miR-6720-5p	miR-8085
miR-769-5p	miR-9500	miR-6734-5p	miR-3180	miR-4716-3p	miR-6721-5p	miR-873-5p
miR-6786-3p	miR-5584-5p	miR-1255b-5p	miR-3187-3p	miR-4722-5p	miR-6724-5p	
miR-4734	miR-4779	miR-1255a	miR-3187-5p	miR-4723-5p	miR-6731-5p	
miR-532-3p	miR-6891-5p	miR-6133	miR-3196	miR-4725-3p	miR-6754-5p	
miR-1539	miR-3173-3p	miR-6130	miR-338-3p	miR-4745-5p	miR-6755-5p	
miR-1224-3p	miR-6834-5p	miR-6127	miR-33a-5p	miR-4749-5p	miR-6760-5p	

Gene:	Alternative name of gene:	Chromosomal location:	Protein:
SLC7A11	xCT	4q28.3	Slc 7A11

miRNA:						
miR-122-5p	miR-3913-3p	miR-489-3p	miR-574-5p	miR-6826-5p	miR-520g-3p	miR-302a-3p
miR-30a-5p	miR-1277-5p	miR-25-3p	miR-3163	miR-498	miR-512-3p	miR-93-5p
miR-148b-3p	miR-5011-5p	miR-367-3p	miR-3941	miR-4279	miR-4640-3p	miR-526b-3p
miR-340-5p	miR-1279	miR-92a-3p	miR-4789-3p	miR-4532	miR-5683	miR-519d-3p
miR-155-5p	miR-595	miR-363-3p	miR-603	miR-1247-3p	miR-520e	miR-20b-5p
miR-128-3p	miR-6867-5p	miR-92b-3p	miR-362-3p	miR-5571-5p	miR-520d-3p	miR-20a-5p
miR-215-5p	miR-4789-5p	miR-190a-3p	miR-329-3p	miR-6778-3p	miR-520c-3p	miR-17-5p
miR-181a-5p	miR-186-3p	miR-223-5p	miR-8485	miR-1281	miR-520b	miR-106b-5p
miR-192-5p	miR-548e-5p	miR-410-3p	miR-6829-3p	miR-5089-5p	miR-520a-3p	miR-106a-5p
miR-32-5p	miR-6835-3p	miR-548t-5p	miR-6791-3p	miR-5589-5p	miR-373-3p	miR-6504-3p
miR-26b-5p	miR-142-3p	miR-548az-5p	miR-1976	miR-4731-5p	miR-372-3p	miR-3653-5p
miR-27a-3p	miR-500a-3p	miR-6874-5p	miR-6747-3p	miR-6506-5p	miR-302e	
miR-19a-3p	miR-767-5p	miR-505-5p	miR-6727-3p	miR-619-5p	miR-302d-3p	
miR-19b-3p	miR-5589-3p	miR-1-5p	miR-4722-3p	miR-150-5p	miR-302c-3p	
miR-218-5p	miR-4282	miR-587	miR-5193	miR-520h	miR-302b-3p	

Gene:	Alternative name of gene:	Chromosomal location:	Protein:
SLC16A1	MCT; MCT1	1p13.2	Slc 16A1

miRNA:

miR-376a-5p	miR-3978	miR-379-5p	miR-3714	miR-563	miR-5691	miR-3681-3p
miR-124-3p	miR-488-3p	miR-3529-5p	miR-548f-3p	miR-380-5p	miR-5006-5p	miR-377-5p
miR-484	miR-6730-5p	miR-4650-3p	miR-548e-3p	miR-6738-5p	miR-3972	miR-513a-5p
miR-376a-3p	miR-5681a	miR-3663-3p	miR-548az-3p	miR-5194	miR-1202	miR-548ae-3p
miR-128-3p	miR-4719	miR-6742-3p	miR-548ar-3p	miR-1914-3p	miR-6864-3p	miR-548ah-3p
miR-615-3p	miR-3936	miR-6852-5p	miR-548a-3p	miR-6761-5p	miR-3672	miR-548aj-3p
miR-320a	miR-3680-3p	miR-939-3p	miR-506-3p	miR-4324	miR-4423-5p	miR-548am-3p
miR-23b-3p	miR-30e-3p	miR-8060	miR-506-5p	miR-6814-5p	miR-6501-5p	miR-548aq-3p
miR-5003-3p	miR-30d-3p	miR-936	miR-892c-5p	miR-4733-5p	miR-431-5p	miR-548j-3p
miR-6074	miR-30a-3p	miR-1972	miR-6866-3p	miR-548m	miR-4294	miR-548x-3p
miR-552-5p	miR-3942-3p	miR-6504-3p	miR-188-5p	miR-3117-5p	miR-3194-3p	miR-6086
miR-374a-5p	miR-6783-3p	miR-4438	miR-7109-3p	miR-425-5p	miR-4755-3p	miR-655-5p
miR-374b-5p	miR-1343-3p	miR-29a-3p	miR-605-5p	miR-4503	miR-924	miR-7162-3p
miR-33a-5p	miR-7154-5p	miR-29c-3p	miR-6124	miR-6792-5p	miR-367-5p	miR-7850-5p
miR-33b-5p	miR-5100	miR-29b-3p	miR-3148	miR-6815-3p	miR-1267	
miR-302a-5p	miR-7151-3p	miR-656-3p	miR-5002-5p	miR-890	miR-302c-5p	
miR-190a-3p	miR-5095	miR-5582-5p	miR-33a-3p	miR-8055	miR-216a-3p	
miR-5011-5p	miR-376a-2-5p	miR-3910	miR-4307	miR-744-3p	miR-27a-3p	
miR-129-5p	miR-4451	miR-5582-3p	miR-576-5p	miR-6805-3p	miR-27b-3p	

Gene:	Alternative name of gene:	Chromosomal location:	Protein:
SLC16A3	MCT3; MCT4	7q25.3	Slc 16A3

miRNA:

miR-16-5p	miR-3074-5p	miR-4519	miR-4740-3p	miR-6747-3p	miR-6825-5p	miR-873-3p
miR-98-5p	miR-3135a	miR-4710	miR-516a-3p	miR-6783-5p	miR-6883-5p	
miR-1226-5p	miR-3616-3p	miR-4721	miR-516b-3p	miR-6785-5p	miR-6886-5p	
miR-149-3p	miR-3677-5p	miR-4722-3p	miR-5693	miR-6804-5p	miR-7160-3p	
miR-2115-5p	miR-4274	miR-4728-5p	miR-6727-3p	miR-6824-5p	miR-7162-5p	

Gene:	Alternative name of gene:	Chromosomal location:	Protein:
SLC16A7	MCT2	12q14.1	

miRNA:

miR-215-5p	miR-4423-3p	miR-20a-3p	miR-759	miR-892c-5p	miR-4748	miR-4464
miR-192-5p	miR-496	miR-4729	miR-506-5p	miR-329-5p		

Gene:	Alternative name of gene:	Chromosomal location:	Protein:
SLC16A8	MCT3; REMP	22q13.1	Slc 16A8

miRNA:

ND

Gene:	Alternative name of gene:	Chromosomal location:	Protein:
SLC16A10	TAT1; MCT10	6q21	Slc 16A10

miRNA:						
miR-21-5p	miR-3926	miR-1825	miR-6504-3p	miR-5698	miR-140-3p	miR-3166
miR-590-3p	miR-6720-3p	miR-3149	miR-942-3p	miR-143-5p	miR-6722-5p	miR-3158-5p
miR-379-5p	miR-3190-5p	miR-6832-5p	miR-4438	miR-6879-3p	miR-122-5p	miR-499b-5p
miR-4451	miR-4428	miR-1273f	miR-4643	miR-4430	miR-1304-3p	miR-509-5p
miR-4650-3p	miR-5195-3p	miR-7151-3p	miR-3674	miR-3652	miR-1281	miR-509-3-5p
miR-3529-5p	miR-145-5p	miR-5095	miR-6758-3p	miR-504-3p	miR-5693	miR-4418
miR-548s	miR-2115-5p	miR-4537	miR-153-5p	miR-3135b	miR-6890-3p	miR-4772-3p
miR-6807-5p	miR-199b-5p	miR-4717-3p	miR-1250-3p	miR-6499-3p	miR-1273h-3p	
miR-566	miR-199a-5p	miR-6746-3p	miR-624-3p	miR-500b-3p	miR-4771	

Gene:	Alternative name of gene:	Chromosomal location:	Protein:
SLC36A1	LYAAT-1; PAT1; TRAMD3	5q33.1	Slc 36A1

miRNA:						
miR-18a-5p	miR-143-5p	miR-381-5p	miR-922	miR-3671	miR-15a-3p	miR-603
miR-500a-5p	miR-3944-5p	miR-767-5p	miR-1226-3p	miR-5704	miR-4717-5p	miR-362-3p
miR-1228-5p	miR-6825-5p	miR-571	miR-4259	miR-6872-3p	miR-3941	miR-329-3p
miR-203b-3p	miR-6729-5p	miR-219a-1-3p	miR-634	miR-4756-3p	miR-6767-3p	miR-8485
miR-3147	miR-4649-5p	miR-4530	miR-6507-5p	miR-1295b-3p	miR-4719	

Gene:	Alternative name of gene:	Chromosomal location:	Protein:
SLC38A1	ATA1; NAT2; SAT1; SNAT1	12q13.11	Slc 38A1

miRNA:						
miR-16-5p	miR-607	miR-4743-3p	miR-5692b	miR-892c-5p	miR-4446-5p	miR-6745
let-7b-5p	miR-340-5p	miR-4639-5p	miR-5692c	miR-31-3p	miR-578	miR-363-5p
miR-30a-5p	miR-374a-5p	miR-6888-3p	miR-369-3p	miR-136-5p	miR-3679-3p	miR-5192
miR-218-5p	miR-5590-3p	miR-374b-5p	miR-4517	miR-4716-5p	miR-6832-3p	miR-4644
miR-320d	miR-142-5p	miR-3148	miR-5583-3p	miR-6868-3p	miR-329-5p	miR-4306
miR-877-3p	miR-570-5p	miR-6124	miR-1277-5p	miR-335-3p	miR-6748-5p	miR-185-5p
miR-484	miR-548ba	miR-215-3p	miR-2113	miR-5096	miR-6880-5p	
miR-570-3p	miR-548ai	miR-6773-3p	miR-889-3p	miR-513b-5p	miR-4483	
miR-6074	miR-548ag	miR-4317	miR-6847-3p	miR-6804-5p	miR-1293	
miR-4726-3p	miR-548m	miR-8076	miR-205-5p	miR-125b-2-3p	miR-6766-5p	
miR-3671	miR-4652-3p	miR-3688-3p	miR-506-5p	miR-4457	miR-6756-5p	

Gene:	Alternative name of gene:	Chromosomal location:	Protein:
SLC38A2	SAT2; ATA2; KIAA1382; SNAT2	12q13.11	Slc 38A2

miRNA:						
miR-30a-5p	miR-3145-3p	miR-301b-3p	miR-1296-3p	miR-148a-3p	miR-1269b	miR-3912-5p
miR-335-5p	miR-4789-5p	miR-4295	miR-4801	miR-455-3p	miR-1269a	miR-548at-5p
miR-124-3p	miR-599	miR-3666	miR-4731-3p	miR-5590-3p	miR-4515	miR-26b-5p
miR-101-3p	miR-4704-5p	miR-6857-5p	miR-212-3p	miR-142-5p	miR-1288-3p	miR-26a-5p
miR-16-5p	miR-1245b-3p	miR-6878-3p	miR-132-3p	miR-4429	miR-4740-5p	miR-181d-5p
let-7b-5p	miR-19a-3p	miR-491-5p	miR-340-5p	miR-320d	miR-4711-5p	miR-181c-5p
miR-18a-3p	miR-19b-3p	miR-4450	miR-199b-5p	miR-320c	miR-6857-3p	miR-181b-5p
miR-193b-3p	miR-130a-3p	miR-6744-3p	miR-199a-5p	miR-320b	miR-4682	miR-181a-5p
let-7e-5p	miR-301a-3p	miR-4757-5p	miR-1825	miR-320a	miR-2355-3p	
miR-3161	miR-130b-3p	miR-194-3p	miR-152-3p	miR-8081	miR-4646-3p	
miR-9-3p	miR-454-3p	miR-4699-3p	miR-148b-3p	miR-4642	miR-4474-3p	

Gene:	Alternative name of gene:	Chromosomal location:	Protein:
SLC 38A3	G17; SN1; SNAT3	3p21.31	Slc 38A3
miRNA:			
ND			

Gene:	Alternative name of gene:	Chromosomal location:	Protein:
SLC 38A4	PAAT; NAT3; ATA3; SNAT4	12q13.11	Slc 38A4

miRNA:						
miR-335-5p	miR-6739-5p	miR-4763-3p	miR-1255b-5p	miR-139-3p	miR-1909-3p	miR-3150b-3p
miR-6858-5p	miR-3153	miR-1207-5p	miR-1255a	miR-6722-3p	miR-4784	miR-6783-5p
miR-4689	miR-6733-5p					
